# In the liminal realm: Qatar's world cup struggle between tradition, modernity, and human rights

**DOI:** 10.3389/fspor.2024.1434522

**Published:** 2025-02-05

**Authors:** Mohammed Al-Thani

**Affiliations:** Physical Education and Sport Science Department, Qatar University, Doha, Qatar

**Keywords:** 2022 world cup, sport mega events, liminality, liminal spaces, human rights

## Abstract

This paper examines Qatar's 2022 World Cup through the lens of liminality, presenting the intricate interplay between tradition, modernity, and human rights. By introducing liminality as an analytical tool, the paper explores how Qatar navigates traditional norms amidst global scrutiny, particularly concerning human rights issues such as migrant labour practices and cultural contestations around human rights. Employing liminality as a conceptual framework, this analysis offers a nuanced understanding of Qatar's endeavour to balance cultural authenticity with international expectations. I reveal why Qatar utilised the transient phase to implement reforms to its social and legal systems, aligning with international human rights while ensuring changes were akin with its cultural identity. The paper highlights the transient nature of change during the liminal phase of sport mega-events, emphasising both the opportunities and challenges presented for changes in Qatar. The paper unpacks Qatar continued navigation in the global stage as an extension of the initial liminal phase, demonstrating its engagements with processes of change and adaptation. The World Cup is a prime example that exposes the dual nature of sport mega events, serving as a springboard for social change, while also revealing underlying cultural and structural tensions. I conclude that Qatar utilised this liminal phase as a rite of passage, enabling the nation to traverse the terrain of modernity while testing the permeability of its cultural boundaries.

## Introduction

Sport mega events (SMEs), such as the World Cup is often celebrated as a beacon of unity among nations. However, beneath this outwardly veneer of international solidarity where nations from all over the globe gather and compete, lie complicated sociocultural forces and disputed narratives. These undercurrents cast doubt on the over simplified interpretation of the events as a single, uniting global event, which unveil a terrain where cultural, social and political tensions collide. This is supported by scholars (1, 2) who critically examine the influence of globalisation on such events. They specifically highlight the World Cup as a reflection and promoter of Western cultural values and ideologies such as democracy, capitalism, individualism, and consumerism. Similarly, Roche (3) conceptualises mega-events like the World Cup as symbolic representations of Western cultural dominance, further illustrating the complexities that underlie the surface image of unity. SMEs not only showcase Western Ideals and influence but carry significant cultural consequences on host nations.

The Berlin Olympics in 1936 known as the “Nazi Olympics” serves as a notorious example where a SME was exploited to display an image of national strength while concealing internal injustices. The Olympic games organised by Nazi Germany were used by Hitler as a medium, for propaganda to exhibit Aryan superiority and his regime worldwide. Although it aimed to promote leniency, human rights abuses were widespread and Jewish athletes were deliberately left out. Despite its stated goal of fostering leniency, there were several violations of human rights, and Jewish sportsmen were specifically excluded. This resonates with Qatar's 2022 World Cup albeit in a dissimilar way, where the event was similarly leveraged to project inclusivity and reform amid critiques of labor abuses and restrictions on human rights. Both events showcases how SMEs create liminal spaces where nations are confronted with global expectations while navigating rooted societal norms. Previous historical events highlight the repeating tensions between human rights and global ideals promoted by SMEs. The World Cup in Qatar is a contemporary case study, unveiling the transitory and challenging nature of change within these liminal spaces.

It is widely argued that sports and sporting events have the potential to foster social change, drive social development, and contribute to broader sustainable development and the advancement of human rights (3). The literature exploring the intersectionality of cultural identities, human rights, and development in the host nation provides crucial insights into how these factors interact and influence dynamics surrounding the World Cup (4). Against this backdrop, this paper specifically examines how a host nation engages with human rights issues and assesses the lasting effects of changes prompted by the event. By analysing the interplay between the World Cup and cultural identities within the host nation, the study aims to illuminate the complex dynamics at play and the transformative potential of such mega-events on societal norms and structures.

Prior research on the World Cup has often used the concept of soft power to explore its connection with national politics, culture, and both regional and international positioning (5, 6). Building upon this framework, the anthropological idea of liminality (7) is used in this research to offer a sophisticated examination of transnational states and the fluidity of identities. Utilising liminality as an analytical lens, this study examines the intricate relationships among the World Cup, societal transformation, and the contested realm of human rights in Qatar. This approach allows for a deeper understanding of how the event both reflects and influences the transitional dynamics within the host nation. Additionally, the implicit tension between maintaining cultural norms and adhering to international standards is a central theme in Qatar's approach to change. The World Cup serves as a poignant example of this tension, representing a liminal state where Qatar grapples with the international expectations and demands associated with hosting a global event. Most existing studies have overused framework such as soft power, nation branding and cultural diplomacy which, while useful incline to overlook or completely neglected the transitory and transient space of such events (8). By focusing on liminality, this paper addresses a critical gap by conceptualising events as “liminal spaces” where societal norms, and global standards are contested, negotiated and potentially redefined. This approach followed in the study revealed complex process of negotiation and adaptations that occur during transitional periods, specifically. In the case of Qatar, where tradition and modernity overlap. Qatar's sociopolitical landscape urges a nuanced approach that balances the preservation of cultural authenticity with the principles of universal human rights. This delicate negotiation underscores the complexities involved in matching national identity with global norms.

## Human Rights and SMEs

SMEs such as the FIFA World Cup and the Olympic Games are often celebrated as promoters of human rights and peace (9, 10). However, research has frequently demonstrated that these events often fail to fulfil their promises, instead eroding the rights of individuals and negatively impacting vulnerable communities (11). The Universal Declaration of Human Rights (UDHR), established in 1948, marked a significant milestone in the discourse on human rights, setting globally recognised standards. Although the declaration does not explicitly mention sports, it unequivocally affirms the inherent dignity and equal rights of all human beings, providing a framework for evaluating the human rights impact of these global events. The early contestation of human rights associated with SMEs can be traced back to the 1968 Summer Olympic Games hosted by Mexico City. This event was intended to celebrate Mexico as a modern, developed nation and to underscore the political significance of the Games. However, in the build-up to the Olympics, a dramatic conflict emerged: thousands of Mexican students took to the streets to protest, culminating in a tragic incident where government forces opened fire, resulting in the deaths of hundreds of students (12). Despite being staged as a showcase of progress and unity; the Olympics were overshadowed by this violent suppression of dissent. More than five decades after this deadly event, the paradox of SMEs continues to persist and may have even intensified. These events are frequently promoted as platforms for peace and human rights, yet they repeatedly face criticism for failing to uphold these very principles, illustrating a deep and ongoing tension between the ideals they espouse and the realities they produce (11, 13).

Scholarly debate surrounding SMEs has expanded rapidly, evolving significantly in focus. Initially concentrated on the financial and economic implications of such events, academic inquiry has broadened to encompass contemporary issues including human rights, environmental impact, climate change, and development (14, 15). This shift followed the United Nations' recognition of sports as a catalyst for development, prompting researchers to scrutinise the actual benefits these events might deliver. Despite widespread advocacy for sports as a means to yield positive impacts on communities—such as promoting human rights and fostering peace—the reality has often proven contradictory. Specifically, nations hosting SMEs find themselves under intense scrutiny, not only regarding the tangible impacts of these events but also concerning their potential to exacerbate human rights abuses and risks (16). The spotlight on these nations raises critical questions about the ethical responsibilities involved in staging such globally watched events and whether the purported benefits are sufficient to offset the social and environmental costs incurred.

As Heerdt articulates, the three critical phases of SMEs—bidding, delivery, and staging— are fraught with potential human rights risks. Each phase plays a vital role in ensuring that the events are conducted in environments that foster the advancement of human rights rather than undermining them. Among these phases, human rights abuses have been particularly prominent during the delivery phase. Recorded violations during this phase have included forced evictions, labour abuses (predominantly affecting migrant workers), suppression of freedom of expression, and the arrest of journalists (17). Such issues spotlight the tensions between hosting large-scale sporting events and maintaining ethical standards in human rights practices. Taking Qatar as a case study, numerous Non- Govermental Organisations (NGOs) have raised alarms over issues concerning migrant workers' rights, freedom of expression, women's rights, and LGBT concerns (4, 18). These highlight the broader implications of hosting SMEs, providing the international community with opportunities to focus attention on human rights issues both within and beyond the sporting context in the host nation. NGOs have been proactive in using SMEs as a leverage point to call out domestic practices. This has been especially evident in Qatar, where the phases of delivery and staging have exposed significant human rights risks and abuses. Such instances underscore a pattern observed in the contemporary history of SMEs, where the grandeur and visibility of these events often bring to light underlying societal and ethical challenges. This dynamic presents an ongoing challenge to reconcile the celebratory nature of SMEs with the imperative to uphold and promote fundamental human rights.

Heerdt (4) delves into the complexities of addressing human rights abuses and holding the responsible parties accountable within the context of SMEs. She highlights the challenges posed by the multitude of actors involved, which adds layers of obscurity to the lines of responsibility and accountability. The presence of various stakeholders—from local organisers and international sporting bodies to governmental entities and corporate sponsors—complicates the attribution of accountability for human rights violations. Furthermore, Heerdt (19) points out that the harms associated with SMEs, whether direct or indirect, create a convoluted landscape that can make addressing these issues seem nearly insurmountable. This complexity is exacerbated by the often chaotic nature of staging such large-scale events, where the rapid pace and immense scale can blur the lines of oversight and enforcement. This convergence of factors leads to a situation where accountability is obscured, complicating efforts to leverage SMEs as platforms for social change. The frequent use of rhetoric around human rights and social transformation in relation to SMEs becomes problematic in this context. The ambiguity surrounding responsibility and the real impact of these events casts doubt on the sincerity and effectiveness of such claims, challenging stakeholders to find more robust mechanisms for ensuring that SMEs truly contribute to positive social outcomes.

Although the work of Heerdt (17) is crucial for paving a clear path towards understanding human rights abuses and minimising risks in future host nations, the present paper diverges into a detailed discussion of how the cultural and socio-political landscape plays a pivotal role in the politics of human rights and change. Moreover, harms that are directly and indirectly linked to the staging of SMEs are described as complex and chaotic, making effective management and mitigation seemingly impossible. This convergence creates a scenario where lines of responsibility and accountability are blurred, contributing to the obscurity that surrounds the continuous invocation of language pertaining to social change and human rights. Additionally, the harms associated with SMEs intensify the liminality experienced by host nations such as Qatar. The potential for transformation that these events purportedly offer is often overshadowed by the messy realities of displacement, labour exploitation, and environmental degradation. These factors not only challenge the integrity of the host but also complicate the broader impact of SMEs on global perceptions of progress and development. This complexity necessitates a more nuanced approach to understanding and addressing the multifaceted impacts of these global events.

Understanding the role of culture is paramount to comprehending the dynamics of human rights and the ensuing changes and reforms in host nations. The intersection of liminality and social change underscores the transformative potential and complexities in navigating cultural identity on the global stage. While Qatar attempts to navigate tradition with modernity, it broach on a journey fraught uncertainty, where decisions, actions and ultimately outcomes is shaped by the liminal space it occupies. In the context of Qatar's cultural landscape as this paper will show the World Cup embodies a liminal space where traditional values converge with global scrutiny instigating social transformations and challenges. A comprehensive approach to assessing changes in human rights must, therefore, consider cultural and social dynamics, recognising both their potential to facilitate change and the barriers they may present. As Qatar navigates this complex interplay of global expectations and local traditions, the World Cup serves as a critical arena for examining these cultural tensions and their implications for social change and human rights reform. This analysis aims to provide a deeper insight into how such SMEs can both challenge and reinforce cultural identities, influencing the trajectory of human rights advancements in host nations.

The aim of this argument is to spotlight the existing tension between Qatar's traditional values and the international scrutiny that has intensified due to its hosting of the World Cup. Qatar's patriarchal structure often conflicts with the new dynamics introduced by such global events. Employing liminality as an analytical tool is particularly pertinent in this context. I argue that the concept of liminality is highly relevant within the realm of SMEs, as these occasions are predominantly about navigating and instigating change. The World Cup, in particular, provides a liminal space where transformative potentials are both obscured and revealed. Through a lens that captures the complexity and nuance of this setting, we endeavour to uncover the hidden truths and transformative potentials enmeshed within the liminal realm of the World Cup, highlighting how it serves as a catalyst for both continuity and change within the host nation.

## Hybridising Human Rights

The process of hybridising rights involves integrating universal human rights principles with the cultural values, norms, and traditions of a specific society (20). Qatar faced a significant challenge in its quest to host a successful and ethically responsible World Cup event. At its core, Qatar's approach to hybridising human rights is about embracing diversity and complexity to foster a more inclusive society where human rights are both respected and tailored to cultural differences. This concept acknowledges that human rights are not a one-size-fits-all solution, as societies interpret rights differently within their unique cultural landscapes. Thus, Qatar's strategy reflects a nuanced understanding of how global human rights standards can be adapted to align with local customs and values, promoting a balanced and context-sensitive implementation of these rights (21).

Giulianotti (22) discusses the Western-centric notion of human rights as a significant obstacle, highlighting its complexity and the hesitancy of non-Western nations to adopt it. He views the Universal Declaration of Human Rights (UDHR) as the foundational document for any discussion on human rights. Although most member states have endorsed the UDHR, enforcing these rights remains a formidable challenge. This difficulty largely stems from the varied political, religious, and cultural backgrounds of states, which lead to diverse interpretations and implementations of human rights (23). Such differences underscore the complex interaction between global human rights norms and local contexts, complicating the universal application of these standards (24).

Griffin's (25) work is pivotal for understanding how Qatar might interpret and apply human rights within its unique cultural context. Griffin (26) references Bhabha's (27) notion of the “third space”, where Qatar has the opportunity to hybridise certain values and norms while considering cultural specificities. The objective is to implement reforms and changes without compromising cultural values. Although this paper aligns with Griffin's (25) argument, it also recognises that certain cultural values and norms are deeply embedded within the social fabric of Qatar, influenced by complex Islamic belief systems.

Acknowledging Bhabha's (28) concept and Griffin's (26) interpretation, this paper expands the analysis by employing the concept of liminality to explore the staging of the event. This approach posits that hosting such an event creates a temporarily contested space where cultural values, practices, and norms intersect with new structures and ideas. The concept of liminality is further elaborated in the following section, setting the stage for an in-depth analysis of the dynamic and contested nature of change instigated by the World Cup in Qatar.

The discourse surrounding human rights in Qatar is not merely about absolute compliance with external values and standards, but rather a nuanced process of reconciling tradition with modernity, ensuring that cultural integrity remains intact. This approach is grounded in the concept that universal human rights principles can converge with local social norms and values (29). However, this process is fraught with challenges. Qatar's approach is often juxtaposed against deeply entrenched cultural beliefs and practices.

The concept of liminality provides a valuable lens through which to examine these tensions. The temporality of the World Cup creates a unique space where traditional norms intersect with new structures and expectations. This liminal space, therefore, becomes a critical arena for exploring how Qatar navigates the complex link between maintaining its cultural identity and adapting to global human rights standards. The authorities in Qatar have adopted a nuanced approach towards inclusivity, balancing their firm conservative values with a unique interpretation of human rights. This strategy intricately intertwines efforts to promote inclusivity while respecting cultural sensitivities. Rooted in Qatar's cultural diplomacy, this approach strives to strike a balance between maintaining its core cultural and religious identity and upholding universal human rights principles. The World Cup serves as a prime example of Qatar's soft power aspirations and illustrates the connections between cultural diplomacy, inclusivity, and the promotion of human rights. By showing a willingness to engage internationally, Qatar aims to capitalise on tangible soft power gains.

Distinct from its predecessors, Qatar's approach encompasses a broad range of initiatives that emphasise values such as social cohesion, inclusivity, equality, and respect for tradition. Notable examples include Generation Amazing a social development initiative, the establishment of an International Labour Office in Doha to enhance workers rights, and the International Centre for Sport Security (ICSS) to promote integrity within sports were all launched in conjunction with the World Cup, underscoring Qatar's commitment to human rights. These initiatives have led to a range of positive outcomes, including advancements in women's rights, increased participation of girls in sports, and improved rights for migrant workers, thereby showcasing Qatar's own understanding of human rights within its cultural context.

Qatar's hosting of the World Cup exemplifies the contested and complex nature of human rights, necessitating political and cultural sensitivities while also exploring opportunities for progress and improvement. The language and discourse adopted by Qatari officials have pragmatically addressed concerns regarding the human rights of migrant workers and LGBT communities. Dissecting discussions of policymakers, organisers, and intellectuals in Qatar, it becomes clear that the concept of human rights is tailored to local understandings of inclusivity, acceptance, and tolerance, and has been brought into sharper focus due to Western scrutiny. Although human rights previously received minimal attention, the global visibility of the World Cup, combined with the proactive involvement of organisers and the government, has positioned it at the forefront of the key aspects of the 2022 World Cup. The intersection of liminality and social change highlights the transformative potential and inherent complexities involved in navigating cultural identity on the global stage. This paper will demonstrate that, in the context of Qatar's cultural landscape, the World Cup acts as a liminal space where traditional values are exposed to global scrutiny, thereby instigating both social transformations and challenges.

## In the Threshold: Navigating the Liminal

The nuances of Qatar's socio-cultural landscape during the liminal phase play a crucial role in shaping the nation's transformation. Transitioning from its primitive roots as a tribal society to rapid modernisation fuelled by natural gas resources, traditional legacies continue to exert a significant influence on society (30). At the forefront are tribal and cultural traditions that contribute to the complexities of societal change (31) Traditional norms and values continue to wield influence, despite the push for modernisation (32). A prime example of these ingrained power dynamics is the Kafala system, where traditional hierarchies have a profound impact on workers' rights and welfare and may impede efforts for reform (33).

One of the key contributions of anthropological studies in sports lies in the understanding of sport as a ritual-like performance with transformative potential, often highlighted by Van Gennep's (1960) concept of rites of passage. Van Gennep introduced the notion of rites of passage as rituals that facilitate the transition of individuals or groups from one social status to another, marking their integration into a new, often higher, social standing. In the transition stage, individuals occupy an ambiguous position, described by Turner (74) as being “betwixt and between”. The purpose of such liminal rituals is to alleviate the potential risks of remaining in a state of perpetual liminality, thus guiding participants through the uncertainty of social transition (74).

Liminality is a period characterized by uncertainty and ephemerality, described as being in a state of betwixt and between (34, 35). The theory originates from anthropological texts used and conceptualised by Van Gennep (36) rites of passage, middle phase of transition signifying “threshold”. In the sports literature, the theory has yet to be fully appreciated and explored, despite the purported claims and uses of sport for social change and transformation. The concept has been widely applied in sport literature, spanning domains like sport events, communal settings, and refugee contexts. Sport events act as liminal spaces for transformation, while at the communal level, sports foster social integration. In refugee contexts, sports aid in healing, social inclusion, and rebuilding identity amid displacement (37). Liminality has been used to examine Sport events transformational outcomes (38), the experiences of marginalised communities in sport for development (SDP) programs (39), and a recent research to assess how top level organisation spaces overlap with grass roots level (40).

Liminality has the potential to create moments of increased global attention and social transformation. The broader applications of the concept lends a useful tool in examining social transformations, and understand where temporary periods of ambiguity leads to new norms and identities negotiation. The dual nature of events, leads to a temporarily distraction of existing norms and potentially usher in long lasting changes in national identity or global perception in addition to fostering tensions between tradition and modernity. The concept of liminality suggests that the transformative impacts of such events might be transient, primarily confined to the duration of the event itself. While Qatar may enact reforms to enhance its international image in the lead-up to the World Cup, there is a risk that these changes could be temporary, potentially being rolled back or remaining superficial after the event concludes and the liminal phase ends.

Moreover, a liminality-focused critique could highlight the limitations of framing changes in migrant workers' rights and women's rights merely as a social legacy. While Qatar may implement reforms in response to international pressure and expectations surrounding the World Cup, the deeper structural issues that perpetuate inequality and exploitation may remain unresolved. Liminality encourages a more nuanced understanding of societal transformation, recognising that the World Cup's liminal space offers both opportunities and challenges for enduring change. The event exists in a complex, rapidly shifting landscape where global political climates and cultural tensions can evolve swiftly. However, the structures guiding such mega-events, like the Qatar World Cup, are slow to adapt, bound by the same limitations faced by other sub-disciplines, such as the top-down vs. bottom-up debate (37). The World Cup is shaped by powerful actors such as Western governments, global corporations and Non-govermental organisations that influence both its staging and broader socio-economic impacts. These structural complexities underscore the difficulty of reconciling modernity with tradition in an event that, by its nature, is a liminal space for both the host nation and global spectators.

Additionally, a liminality-focused analysis might explore the tensions between Qatar's aspirations for modernisation and its preservation of patriarchal and traditional norms. While Qatar may strive to project a progressive image through reforms in migrant workers' rights and women's rights, these changes could be limited by entrenched cultural attitudes and power dynamics. This approach prompts a thorough examination of the complexities of societal transformation, acknowledging the ongoing interaction between tradition and modernity within Qatar's evolving social landscape.

The World Cup is the epitome of liminality, which acts as a disruptive force within the host nation that invades and potentially bypass traditional societal structures and norms, shaking up the status quo and existing social order. Within the liminal phase, ingrained social institutions are confronted, revealing the nexus between modernisation, tradition and social change. This seismic shift within the social structure in Qatar, presents opportunities for social change but has its own challenges and risks as normalised old structures are disturbed. As shown in Qatar, structures that controlled migrant workers have been disrupted, causing human rights consequences. The implications of the liminal phase caused by the World Cup are profound on populations, communities, and individuals in multifaceted ways, reshaping societal norms and expectations during this transitional period.

Liminality engenders uncertainty and instability in society as it becomes the site of intense clashes of ideas. The bounded duration of events, along with preparations, reforms, and increased scrutiny, amplifies these circumstances. During such significant transitions, liminality fosters a heightened sense of uncertainty and apprehension, as Qatar undergoes social transformations connected to the event. This paper focuses on the social transformations within the dynamics and cultural norms of Qatari society. The exposure to diverse perspectives serves as an example of the wave of ideas, values, and experiences to which the country is exposed. The influx of international visitors, people of different backgrounds, sexual orientations, and the global spotlight on the event create opportunities for greater tolerance and acceptance within society, ultimately challenging orientalist views about Qatar (36). However, the liminal phases also harbour tensions within Qatari society that can be exacerbated, as this paper elucidates. A significant example of this is the social disparities and inequalities, notably the exploitation of migrant workers. These issues highlight the complex interplay of change and resistance within the context of liminality, underscoring the multifaceted impact of hosting such a major event.

The liminal space in Qatar, exacerbated by hosting the World Cup, teeters between tradition and modernity, revealing the friction between Qatar's conservative values and Western ideals. The juxtaposition of these worlds—tradition and modernity—has surfaced contentious subjects and practices. Qatar's conservative societal norms, particularly regarding LGBTQ+ rights and, to a lesser extent, gender equality, often clash with Western standards (35). These existing tensions are further intensified by the scrutiny and criticism of external observers. From a human rights perspective, the physical spaces of construction sites become contentious zones where labour exploitation emerges. Thus, the transformation of Qatar's landscape not only represents physical change but also captures a liminal space intersecting with critical human rights issues. This dynamic illustrates the complexity between maintaining cultural identity and global expectations, highlighting the challenges and pressures faced in such transitional phases.

The liminal phase becomes a battleground where traditional values and Western ideals clash, and national identity converges with the demands of the international community. Qatar's patriarchal societal structures and deeply ingrained cultural norms present significant challenges to the advancement of human rights as promoted by international NGOs, sports federations, and the broader international community (40, 41). These entrenched cultural frameworks resist and contest changes, particularly in how rights are understood and applied from a Western perspective (42). This contention highlights the complex dynamics at play as Qatar navigates the pressures of aligning with global human rights standards while maintaining its cultural integrity.

The liminal phase exerts a profound impact on the social fabric of Qatar, particularly during the accelerated pace of change brought on by the World Cup. This period of transition can lead to impermanence and instability as individuals, organisers, and the government navigate societal shifts. The scrutiny from the international community brings various pressures that manifest in different effects and outcomes for individuals. A primary example is the infringement on migrant workers' human rights, which fosters uncertainty, stress, and unease. Beyond the individual impacts, these issues also contribute to broader societal unrest, as evidenced by protests among migrant workers (43). The temporary nature of this liminal phase is reflected across various segments of society, highlighting the transient yet intense disruption that accompanies such major events.

## Negotiating Tradition and Modernity: Qatar's Liminal Journey Through the World Cup as a Catalyst for Cultural and Social Transformation

Qatar stands at the crossroads of tradition and modernity, navigating a complex terrain that involves balancing the promotion of inclusivity with the preservation of cultural values and human rights. The World Cup has set the nation on a path to confront these challenges, highlighting the importance of adopting global standards of inclusivity and human rights. As a transnational global event, the World Cup possesses an unparalleled capacity to transcend boundaries, facilitating the flow of foreign ideas and human rights principles into previously unreached regions. This dynamic not only challenges existing norms but also offers opportunities for substantial cultural and social advancements in Qatar. Beneath the guise of progress lies a complex web of tensions. Qatar's societal structures and cultural norms are challenged by the ideals promoted by the Western world. The influx of foreign ideas, driven by the World Cup, international media, and NGOs, has amplified these tensions, compelling Qatar to confront uncomfortable truths about its norms and practices. For Qatar, it was crucial to navigate the delicate balance between tradition and modernity. The modernisation process is often seen as incompatible with the rigidity of tradition. This dichotomy, between preserving cultural identity and adhering to global human rights standards, places Qatar at a crossroads, grappling with the complexities of its own identity.

Qatar has adopted a flexible and adaptive approach to dialogue and engagement with the international community, addressing criticisms through substantive reforms (26). This strategy reflects Qatar's aspirations not only to achieve an acceptable international image but also to play an active and significant role both politically and in the world of sports (28). Consequently, Qatar is compelled to address the social ramifications of these global engagements, striving to reconcile its deep-rooted traditions with the demands of a rapidly changing world (44).

Qatar's soft power aspirations have shaped a cultural diplomacy approach that does not unconditionally accept Western ideals but rather displays a degree of leniency to both outward audiences and inward visitors (45). Qatar's cultural diplomacy efforts aim to capitalise on its soft power influence, with a strategic focus on promoting inclusivity and human rights to position itself as an advocate of tolerance and inclusivity on the international stage (46). Critics often argue that this strategy serves to whitewash Qatar's questionable human rights record, particularly concerning labour exploitation (24, 47).

Qatar remained stern in protecting its cultural and religious traditions. The country's approach to alcohol restrictions, dress codes, and public expressions of political identity demonstrated a commitment to uphold its Islamic values. For Qatar, this approach was more than a mere position of defense: it was a conscious declaration of sovereignty and cultural identity in face of global scrutiny. By showing commitment to preserving these values, Qatar reinforced its role as a guardian of Islamic culture while asserting its right to define the terms of its engagement with the wider world. This balancing act highlights the tension inherent in Qatar's liminal phase. While the World Cup granted space for the experimental application of modernity, the nation drew firm red lines around anything seen as non-negotiable. These boundaries signified to Qatar that modernization did not have equate to the uncritical adoption of Western norms but rather a selective and purposeful process that complemented its cultural ethos.

The concept of liminality serves as an important concept to discern and unpack Qatar's motivations behind reforms and engagement with human rights in the build-up to the World Cup. Liminality is underscored by ambiguity and uncertainty, creates a space where Qatar can experiment changes while setting boundaries that align with its cultural and religious values, ensuring that the changes remained contextually grounded and reversible if necessary. Thus, Qatar's reforms are not merely reactive but reflect a calculated effort to manage the tensions between tradition and modernity, utilizing the temporary ambiguity of the World Cup's liminal phase as a testing ground for balancing global acceptance and local authenticity. and uncertainty, creates a space where Qatar can experiment changes without committing to change in the long run. Purposefully, Qatar's used the World Cup liminal phase to project an image of modernity and reform while navigating its deeply rooted tradition, despite of challenges and disturbance to its social fabric and identity. One way to interpret the “why” behind Qatar actions is a calculated reaction to global expectations, capitalising on the event transitory nature to briefly conform to international standards. Qatar sought for soft power gains, counter orientalist narratives about the country by enacting changes and scaling its inclusivity measures.

Many accusations of whitewashing fail to appreciate the complex intricacies of Qatar's sociopolitical landscape. Critics often claim that developments in human rights are merely used as tools to enhance the country's international image. However, tangible evidence suggests otherwise, as Qatar has engaged in extensive reforms to improve the rights of migrants and women (23, 48). These reforms, despite encountering some challenges during their implementation (49), indicate a significant shift towards addressing these critical issues. This proactive approach underscores Qatar's commitment to reconciling its international image with its domestic policies, striving to transform its global perception through genuine social and legislative changes. Efforts to address labour rights issues, in particular, demonstrate Qatar's commitment to substantive changes (50). Despite these initiatives, the enduring tensions between tradition and modernity persist, underscoring the complexities of Qatar's socio-political environment. These dynamics are further complicated by the liminal space Qatar occupies, as it navigates the pressures of upholding traditional values while adapting to global human rights standards. This situation highlights not only the challenges but also the potential pathways for Qatar to reconcile its rich cultural heritage with contemporary global expectations.

## Transcending boundaries, The liminality of Kafala abolishment

Liminality as mentioned earlier refers to a state of ambiguity and transition which is foregrounded by thresholds or borders between different states. In this context I propose the process of abolishing Kafala system, a social reform and reconfiguration within the Qatar social an legal framework represents and marks a liminal period, where traditional boundaries and structures are challenged arising challenges and risks.

Current configurations of the kafala system are concomitant with exploitative practices and are widely conceptualised as modern forms of slavery. In Qatar, all foreign workers must be sponsored by their local employer who must issue an exit permit if they wish to leave the country, whether this is on a permanent or short-term basis (51). The sponsor is then required by law to return the passport of the sponsored individual upon completion of the necessary procedures. This remains the case for sponsored individuals who wish to renew their residence permits. These conditions clearly contravene legal stipulations concerning the freedom of movement as encapsulated in the Universal Declaration of Human Rights, as well as the International Convention on the Elimination of All Forms of Racial Discrimination (52). Prior to the current reforms, guest workers in Qatar were further mandated by law to produce no objection certificates or NOCs from their employers if they wished to change jobs before their contracts expire, although if workers do not wish to leave the country immediately, NOCs are required upon expiration of the contract. A key tangible statement of reform was the establishment of the International Labour Office in Doha in 2018. The focus of the ILO is to reform Kafala, facilitate and streamline acceptable international labour standards and convention through cooperation with the state of Qatar. In addition, to align reforms with international labour laws the office opening was set to go beyond current reforms and sustain a lasting and positive legacy of the 2022 World Cup.

In the liminal terrain, migrant workers and their communities encompass a landscape fraught with uncertainty and peril. Abuses of power in their work place require protective enforcement mechanisms to create a safe environment for workers advocacy and speaking up against power structures and systemic oppression. However, such stance can come at a cost of harsh reprisals to preserve the status quo. As Alkhayareen (53) argued that the current labour environment and the absence of trade union continues the vulnerable position of the migrant worker population. In the study, he found out employers or business are still confiscating passports, stating a lack of enforcement mechanism to prevent confiscation, In addition, workers are abused by withholding of salary. Power asymmetries within this liminal terrain remain as structures behind the Kafala system are not instantly eradicated due to recent reforms. The liminality here involves a societal and political dimension in which traditional view of hierarchy and paternalists employer-employee relationships are not necessarily ceasing to exist to due change in legal frameworks. The process of reforms must consider underlying cultural currents by seeking to transform employers exploitative mindsets.

In addition, economic factors influence this liminal period in a construction industry where profit is prioritised over labour protections and rights. The construction industry is dominated by billion-dollar companies that are unlikely to welcome new regulations and restrict their power over migrant workers and worker camps. Given the nature of the labour system, it offered a stable, if deeply imbalanced, framework that allowed employers to secure a consistent influx of low-cost, short-term migrant workers. Within this grave environment, efforts to create a more equitable labour system can be enduring to implement in practice due to the sheer number of workers. The liminal realm created by the World Cup, intensified by global exposure, disrupted this stability. The increased visibility of labour abuses, newly enforced reforms, and international demands challenged that previous state. However, in this transitional state, the old system of Kafala has not been completely eradicated, nor have migrant workers rights been fully realised. Companies now find themselves in an uncertain landscape where reforms challenge their normalised authority over workers conditions. As a result is a disruption to the old structure, wherein economic incentives lie at the heart of this tension. This disruption has prompted reforms of labour practices initiated by the State of Qatar, pushing companies to adopt more humane practices in their labour force. Nonetheless, the path to genuine reform remains fraught with obstacles, as traditional power dynamics continue to shape the construction industry and migrant workers in Qatar.

## The Impact of Patriarchal Norms on Migrant Labor Practices: Unveiling the Dynamics of “Doxic” Relations in Qatar

Qatar's societal framework, characterised by patriarchal structures and deeply ingrained cultural norms, presents significant challenges to the promotion of human rights as understood via Western-centric lens (54). These entrenched cultural frameworks not only resist but also contest changes in many aspects of Western conceptions of rights and their applicability. The enduring practice of the Kafala system exemplifies how longstanding social stratification can exacerbate the vulnerabilities of marginalised populations, particularly migrant workers (55). This sponsorship system binds the legal status of migrant workers to their employers, creating an imbalance of power that often leads to exploitation and abuse (56). This arrangement highlights the systemic vulnerability of such groups, making it difficult to implement reforms that align with international human rights standards. Furthermore, the interaction between Qatar's traditional practices and international human rights norms raises complex questions about sovereignty, cultural relativism, and the universality of human rights. The challenge lies in navigating these cultural and legal discrepancies to foster a more inclusive approach to human rights that respects Qatar's cultural identity while also adhering to global standards. This ongoing tension underscores the need for a nuanced understanding of cultural contexts in the implementation and advancement of human rights globally.

The notion of “doxic relations”, rooted in patriarchal and traditional norms, perpetuates cycles of exploitation and discrimination (57). Migrant labourers, classified as “dox”, are subject to systemic inequalities that maintain their marginalised status, Within doxic relations, workers are placed in a system that normalises and perpetuates their subjugation shaped by unspoken societal norms and power dynamics. In the context of migrant labour practices, the intersections with racial and ethnic identity further amplify existing power imbalances, hindering the advancement of human rights (58). This hierarchical structure is reinforced by dominant values that favour the interests of power elites and dominant groups, often overlooking the rights and well-being of migrant communities. In Qatar, social stratification is pronounced, with power dynamics heavily influenced by nationality, ethnicity, and socio-economic status (59). Historically, migrant labourers occupy the lowest rungs of this hierarchy, making them particularly vulnerable to systemic discrimination both in the workplace and in broader society (60).

The Kafala system exemplifies how these entrenched hierarchies operate, binding migrant workers' legal status to their employers and creating a dependency that facilitates exploitation. This system not only restricts the freedom and rights of migrant workers but also perpetuates their marginalisation within Qatari society (61). Efforts to reform these practices face significant resistance due to the deeply embedded cultural and socio- economic structures that benefit the dominant groups (62). The role of citizens' attitudes towards low-skilled migrants is also worthy of note as ambivalent attitudes about foreign workers trickle down to inform how they are treated. Diop et al. (50, 63) which have drawn from nationally representative surveys in Qatars have provided significant insight into the citizens' attitudes towards migrants. The study found that the sample of citizens featured in the study conceptualised foreign workers as making positive contribution to the development of their country however, concurrently, they expressed concerns about the impact of migrants on national resources with a focus on health systems and the economy (63). Furthermore, the majority of citizens also prefer the current kafala system to be maintained.

The doxic situation perpetuates a status quo where certain labour practices are normalised despite their human rights implications. Constraints to change arise from this liminal space, where entrenched interests and beliefs push back against reforms. Traditional notions inhibit migrants' ability to advocate for and demand meaningful change. The perpetual traditional notion of social hierarchy within this liminal situation contributes to a sense of uncertainty and ambiguity. In such an ephemeral and turbulent environment, stakeholders may act more leniently and prioritise maintaining the status quo, as disrupting it poses risks to social stability.

## Cultural contestation: LGBT and Qatar

LGBT rights were inevitably a contentious issue during the 2022 World Cup in Qatar (64). The complexity of LGBT rights in this context arises from the domestic laws and cultural norms in Qatar. While the focus on LGBT rights is not exclusive to Qatar, it is particularly significant in the context of SMEs (58, 65).These events have often been leveraged by civil rights movements, including LGBT communities, to protest and mobilise for mass movements.

In Qatar, bisexuality and homosexuality are criminalised by law, with any expression or act being condemned. These laws are rooted in Islamic beliefs, which strictly forbid homosexuality (55). The legal framework in Qatar is deeply intertwined with these religious principles, leading to unconditional public condemnation and severe backlash against any LGBT activities. Discourse on LGBT rights in Qatar has been minimal, with little mention of their rights (26). The acceptance of LGBT people arriving in Qatar to attend the World Cup has been a significant concern. This prompted the Emir of Qatar Sheikh Tamim Bin Hamad, to take a firm stance on the acceptance of any community, including the LGBT community in Qatar. Nonetheless, the LGBT rights movement has brought domestic issues concerning LGBT rights in Qatar to the forefront. The absence of LGBT rights and the fears of LGBT individuals in Qatar are major concerns (66).

The LGBT issue exemplifies the clash between tradition and modernity, highlighting the intensified liminality it triggers. The remarks of the Head of Security for the 2022 World Cup, Al Ansari (67), underscore the rigidity of current social structures. He stated, “We would not change our religion for 28 days”, reflecting the unyielding stance on maintaining traditional beliefs. The discussion surrounding LGBT rights and public expressions of sexual orientation in Qatar presents a complex interplay between law, cultural practices, and international expectations. While LGBT acts in public domains are strictly prohibited by law in Qatar, the enforcement of these laws is not always consistently applied (68). This discrepancy highlights the nuanced application of a hybridity lens and a relativistic approach to human rights, suggesting that Qatar has developed its own ideal type of practice.

Qatar's approach to hosting international events, such as the FIFA World Cup, illustrates this complexity. On the one hand, the country promotes inclusivity and extends a welcome to all, regardless of sexual orientation, belief, ethnicity, or nationality. On the other hand, this inclusivity is framed within local cultural understandings and norms. The global media spotlight on Qatar leading up to and during the event brought significant attention to the issue of LGBT acceptance, with concerns about how LGBT visitors would be treated.

Qatar approach to human rights and inclusivity created a distinct liminal space highlighted by transition and ambiguity, where cultural norms and international expectations collided together. In spite of Qatar critical stance on LGBT, the nation adopted a stance that showed to attune global norms in addition to withstanding certain boundaries reflective of local laws and values. LGBT identification were not permitted within the stadium vicinities highlighting a boundary within this liminal space. This prudently maintained balance corresponds Qatar's transitional role as it navigated challenges of global scrutiny against its own socio-cultural framework. In this way, Qatar World Cup is viewed as a liminal experience for the nation where its identity as a conservative society intersects with its role as a global event host.

By occupying this liminal position, Qatar created a transient reality where certain boundaries of acceptance were expanded yet contained shaped by demands of international community and local laws and values. This liminal space did not eliminate cultural or ideological differences; on the contrary, it provided a context wherein the meeting, conflict and negotiation of identities, values, and practices occurred. For example, the ban on LGBT symbols around the stadiums should not be interpreted solely as hostility towards LGBT rights but rather as an assertion of Qatar's cultural limits in what was a worldwide, yet distinctively Qatari setting.

Despite these concerns, Qatar maintained a strong stance on the acceptance of all individuals. The Centre for Sport and Human Rights published a report on human rights practices during the event, indicating that there were no reported incidents of discrimination or violence against LGBT attendees (69). This report suggests that, at least during the event, Qatar was able to balance its local laws with a degree of tolerance and inclusivity expected by the international community. The absence of reported incident may reflect Qatar successful event management and its commitment to ensure the respect and safety of all visitors. While the report offers a positive assessment of the tournament inclusivity, it is essential to situate it as a larger discourse on the performative aspects “transient state” of change during the World Cup.

The response by the Qatari authorities followed a meticulous approach to promote inclusivity for all, while deliberately avoiding the integration of Western values associated with the LGBT movement. The position of people in power such of the Emir mirrors broader cultural and political efforts of inclusivity measures due to global expectations. This approach is embedded within a schematic framework that reflects Qatar's unique stance on human rights, which is inextricably linked to its cultural diplomacy. Qatar navigates a fine line between tolerance and intolerance, balancing the promotion of inclusivity with adherence to domestic values.

This strategy is endorsed at the highest levels of government, with both the national head of security and His Highness the Emir of Qatar supporting the approach. By doing so, Qatar aims to project an image of openness and acceptance, while simultaneously preserving its cultural and societal norms. This dual approach underscores the complexity of human rights practices in Qatar and highlights the country's efforts to reconcile international expectations with local traditions.

## Reflections on Transition, Implications, and Pathways Forward

I Alongside the liminal exploration that offers a temporary setting for cultural renegotiation and societal change, the event's aftermath emphasises the effects of these changes. Host nations of SMEs constantly face post-event hurdles in sustaining legislative reforms as the fleeting momentum and space for reforms, within the liminal realm fade, along with a decline in political and public will. In Qatar, the politics and laws surrounding migrant labour have come to the forefront of human rights concerns SMEs possess a significant force that can serve as a catalyst for initiating change within the host nation's political and social structures. In Qatar's case, the attention and scrutiny brought by such events can help address long-standing issues related to migrant labour rights, pushing for reforms that might not have been as vigorously pursued otherwise (70). The transformative potential of events emphasise the important to capitalised such space, offering room for lasting societal improvements.

Qatar willingness to integrate its local values with international scrutiny sets a precedent to nations seeking to leverage global events to channel their soft power. The paper offers implication for policy making: government can use liminal spaces to project inclusivity and tolerance while preserving their cultural distinctiveness. However, ensuring the authenticity and sustainability are integral to avoid performative compliance and superficial actions. For Qatar, the experience underscores the need to institutionalize some of the temporary accommodations made during the World Cup to strengthen its long-term reputation as a tolerant and globally integrated nation.

Qatar harnessed the World Cup to accentuate its global diplomacy by leveraging the liminal spaces created from hosting the World Cup. It allowed a space to project inclusivity and modernity while safeguarding cultural distinctiveness, underscoring the strategic leverage of liminal spaces. For nations hosting mega-events, the spaces created present opportunities to project inclusivity and modernity while safeguarding cultural distinctiveness. Qatar's success in managing international expectations, despite its limitations, highlights the importance of carefully calibrated policies that align local realities with global aspirations. Such events can provide host states with an opportunity to try out more advanced changes in controlled situations, gaining good will and soft power without completely losing their cultural integrity. However, the transient nature of these liminal spaces highlights the necessity of sustainable reforms and improvements. This is demonstrated by Generation Amazing, which promotes inclusivity, cross-cultural communication, and societal advancement while acting as a long-lasting, significant legacy of the World Cup transition through it sustainable development efforts (71). The position of Generation Amazing is an indelible mark of the liminal World Cup phase longevity through encompassing openness and tolerance reflecting Qatar overarching strategy within its governance framework. Facilitators of the first trial phase, like Generation Amazing, are prime examples of the continuous efforts to further the goals and improvements set forward during the World Cup.

In the aftermath of the event, migrant workers face a slew of obstacles, the most significant of which is uncertainty. This uncertainty is exacerbated by the transient nature of the contractual jobs that workers receive upon their initial arrival in the country. The temporary nature of their jobs that workers are given exacerbates this uncertainty. Workers are frequently left in a tenuous limbo because most contradiction contracts cease at the conclusion of the even (72). Figuratively and temporally, they are cast into the unknown, as their fragile jobs are bound to short periods with minimal opportunities to secure long- term employment.

The manifestation of a liminal process experienced in the World Cup calls for a rethinking of legacy beyond static predefined frameworks. Legacy is often encapsulated in terms of tangible, intangible impacts derived from preordained aspects. However, this papers highlights that legacy is influenced and shaped by the performative and transitional dimensions of liminal spaces which exhibits itself in such events. The World Cup created a temporary yet meaningful platform for dialogue, contestation, potentially shaping its global identity and influencing its cultural diplomacy in the long term. Policy makers and organisers should establish that events are not isolate performances but mutable moments presenting opportunities to achieve goals such in Qatar case soft power aspirations and reforms. In Qatar, the tolerance during the World Cup should not faze out after the liminal space closes which requires commitment to domestic polices, strengthening the nations global standing over time.

As Burnett (73) noted, racial divides based on colour, race, and background persisted in South Africa as a legacy of the apartheid era, despite robust efforts to dismantle them during the hosting of major events. The author argued that deeply ingrained social structures require more than just policy changes to shift. Similarly, in Qatar, meaningful progress will necessitate a coordinated effort that integrates reforms across multiple dimensions of society. This comprehensive approach is essential for fostering a more inclusive and equitable environment for all residents, particularly migrant workers.

Furthermore, as unpacked in the liminal realm, at the heart of Qatar approach to change was to manage the inherent tension between culture norms and international standards. Qatar struggled to satisfy worldwide expectations and the demands of hosting a global event during the World Cup, which served as a potent illustration of this tension. Given Qatar's sociopolitical environment, a nuanced approach that considers the intricate difficulties of upholding universal human rights ideals and preserving culture is necessary. As Qatar aims to preserve to its cultural authenticity, international scrutiny and pressure demand that it uphold global human rights standards and soft power aspirations. The tension achieving its threshold within the liminal phase highlights the need for a sophisticated approach that would allow the perseveration of cultural identity and respect for the values and human rights norms. For Qatar to achieve this delicate balance requires ongoing attention and negotiation as it navigates the challenge of aligning its domestic policies with international expectation without compromising its cultural identity.

A key takeaway for future host events is the importance of establishing clear protections and commitments within a common understanding of acceptable practices. To prevent abuses, such as those faced by migrant workers in Qatar, FIFA must ensure that minimum criteria are met by host nations. In Qatar, issues surrounding migrant labour practices were deeply ingrained long before they were brought to international attention. Furthermore, there needs to be post-event follow-up by FIFA and NGOs to ensure that the positive legacy of the event is sustained. Tangible changes, for instance ILO office, are examples of legacies that should be harnessed and leverage to realise migrant workers human rights. The concept of liminality suggests that there are both constraints and facilitators to change and reform implementation. In Qatar, these constraints often stem from deeply entrenched doxic social and cultural practices. Addressing these challenges requires ongoing oversight and engagement to promote lasting improvements in human rights and labour conditions.

## Conclusion

In conclusion, the liminal phase represents a critical juncture in Qatar's contemporary history, where tradition intersects with modernity, and societal transformation hangs in the balance. By navigating this transitional space with strategic foresight, Qatar is shaping a legacy that reflects its national identity and conservative values while engaging with the global community. Qatar's subtle approach to reconciling universal values with cultural norms demonstrates a delicate tactical interplay. The country seeks to create moments of inclusivity and tolerance, even if these efforts appear fleeting. Liminality, as a period, presents uncertainty and disruption, challenging societal norms and power dynamics. The response and adjustments made during the delivery stage of events, such as the World Cup, showcase Qatar's adeptness at managing the uncertainties that arise in this transitional space.

Qatar's efforts highlight the importance of a nuanced approach in navigating the complexities of cultural preservation and international expectations. The country's ability to maintain its cultural authenticity while addressing global human rights standards reflects a sophisticated strategy. This balancing act is crucial for fostering an environment where societal transformation can occur without losing sight of traditional values. Moreover, the implications of this liminal phase extend beyond Qatar. Future hosts of SMEs can learn from Qatar's experience, understanding the necessity of cultural appreciation and the importance of ongoing engagement and oversight. Ensuring that minimum criteria for human rights and labour conditions are met, and following up post-event to sustain positive legacies, are essential steps for any host nation.

Moving forward the lessons learned from Qatars journey provide insights, for people and groups involved in community development initiatives. By understanding the paths between tradition and modernity and using the knowledge gained from this phase stakeholders can plan a direction towards a more inclusive and fair transformation of society. Whether working at the level or in high level policy discussions recognizing the subtleties of transition can guide effective approaches to navigating the complexities of cultural evolution. Ultimately it is the communication and relationships built within this phase that will shape the outcomes of community development efforts, in Qatar and beyond. By embracing the flexibility of transition and recognizing how societal forces are interconnected individuals and groups can collaborate towards creating an more future for everyone.

Existing studies view sport events as having lasting impacts on the host nation. However, they fail to capture the ephemeral nature of events as presented and discussed in this paper. The concept of liminality brings this transitory nature into focus, questioning whether reforms introduced in the build-up to the World Cup are sustainable or superficial. By framing the event as a liminal phase, this paper contributes to the theoretical debate on whether mega-events truly drive long-term change or merely create temporary moments. People and groups involved in efforts to end the Kafala system regardless of where they stand on the spectrum of advocacy and action can find value in this conceptualisation of liminality. Those actively participating in movements can use this idea to pinpoint ways to influence higher level decision making. Top tier organizations and policymakers can learn from the insights and experiences of those working directly on these issues to shape down policies and project. By bridging the divide, between grassroots initiatives and institutional structures they can ensure that policies are rooted in the challenges faced by workers and address the diverse needs of affected communities.

Essentially although liminality exhibit possibilities for change, I underscore the obstacles that hinder progress in Qatar specifically concerning the World Cup. By delving into the nuances of liminality and its impacts throughout society, the paper offers an understanding of the challenges to bringing about change, amidst entrenched power dynamics and vested interests. Qatar's navigation of this liminal space underscores the potential for meaningful change when cultural understanding and global standards are thoughtfully integrated. Importantly, the why behind Qatar's reforms as a result of the World Cup can be understood through the application of liminality. The transitional phase provided Qatar with a rare opportunity to engage in a strategic fluid space embracing certain global norms to enhance its soft power while carefully maintaining boundaries akin with its cultural identity. By leveraging the liminal space formed by the World Cup, Qatar strived to position and channel itself as a modern, forward-thinking nation actively engaging with international expectations without abandoning its tradition and cultural integrity. The country's journey through the liminal phase serves as an example to the possibility of harmonising tradition with progress, creating a World Cup legacy that echos both locally and internationally. Beyond the basic concept of a balancing act, liminality is a symbolic negotiation process in which interactions with the international community create human rights responses. In addition to being a venue for change, the World Cup serves as a rite of passage for Qatar as it examines its place in the international system and assesses how permeable its cultural barriers are to criticisms of human rights.

## Data Availability

The original contributions presented in the study are included in the article/Supplementary Material, further inquiries can be directed to the corresponding author.
